# Factors Related to Fetal Death in Pregnant Women with Cholera, Haiti, 2011–2014

**DOI:** 10.3201/eid2201.151078

**Published:** 2016-01

**Authors:** Erin Schillberg, Cono Ariti, Lindsay Bryson, Rodnie Delva-Senat, Debbie Price, Reynold GrandPierre, Annick Lenglet

**Affiliations:** Médecins Sans Frontières, Port-au-Prince, Haiti (E. Schillberg, L. Bryson, R. Delva-Senat);; London School of Hygiene and Tropical Medicine, London, UK (C. Ariti); Médecins Sans Frontières, Amsterdam, the Netherlands (D. Price, A. Lenglet);; Ministère de la Santé Publique et de la Population, Port-au-Prince (R. GrandPierre)

**Keywords:** fetal death, cholera, bacteria, enteric infections, pregnancy, Haiti

## Abstract

We assessed risk factors for fetal death during cholera infection and effect of treatment changes on these deaths. Third trimester gestation, younger maternal age, severe dehydration, and vomiting were risk factors. Changes in treatment had limited effects on fetal death, highlighting the need for prevention and evidence-based treatment.

Cholera infections during pregnancy are associated with high rates of fetal death, especially when women are severely dehydrated (*1**–**7*). In Haiti in 2011, pregnant women with clinical signs of cholera who sought treatment from Médecins Sans Frontières (MSF) in Port-au-Prince were sent to a general cholera treatment center (CTC). In April 2012, MSF established a CTC to improve fetal outcomes in pregnant women by facilitating intensive follow-up for dehydration and rapid access to obstetric and neonatal services. In June 2013, a more aggressive rehydration protocol was implemented (Technical Appendix Table 1). To assess the effects of cholera infection, establishment of a specialized CTC, and the new rehydration protocol, we conducted a retrospective cohort analysis of pregnant women with suspected cholera admitted to MSF’s CTCs during September 1, 2011−December 31, 2014.

## The Study

This study was approved by the National Bioethical Committee of the Ministry of Public Health and Population of Haiti. A cholera case-patient was defined as someone who passed >3 liquid stools with or without vomiting or dehydration in the previous 24 hours or within 6 hours of seeking treatment. Women of childbearing age were asked whether they were pregnant. Urine dipstick tests were conducted in cases of uncertainty or early stages of pregnancy. Gestational age was determined by date of the woman’s last menstruation, uterine height, or ultrasound. Fetal status was assessed at admission and hourly by using fetal stethoscope or ultrasonic fetal Doppler. Women who had a miscarriage at home and were not bleeding at admission were not classified as pregnant. Dehydration status was determined according to World Health Organization categories (Technical Appendix Table 1) (*8*). We assigned women into 3 treatment groups (TGs) according to whether they were treated in the general or specialized CTC and whether they were given the original or new protocol (Technical Appendix Table 1).

We analyzed fetal outcome for all pregnant women by initial signs and symptoms, TG, and clinical evolution. Multiple logistic regression modeling was used for adjusted analyses. All analyses used Stata software version 12.0 (StataCorp LP, College Station, TX, USA).

During September 1, 2011−December 31, 2014, a total of 936 pregnant women were admitted. Thirty-six were excluded from analysis: 33 (0.35%) lacked fetal outcome data, and 3 (0.03%) died (1 each during second and third trimester; for 1, trimester was unknown). Of the remaining 900, mean age was 26.7 years (range 15–48, median 26 years); 168 (18.9%) were in their first trimester, 303 (34.2%) second trimester, and 416 (46.9%) third trimester. Trimester was unknown for 13. A total of 444 (49.3%) sought treatment within 24 hours of symptom development.

Fetal death occurred in 141 (15.7%) of the 900 analyzed pregnancies, more often in women <20 years of age (odds ratio [OR] 2.0, 95% CI 1.2–3.2), in their third trimester (OR 2.4, 95% CI 1.4–4.3), seeking treatment >24 hours after symptom onset (OR 1.5, 95% CI 1.0–2.2), with severe dehydration (OR 2.2, 95% CI 1.2–3.8), or who vomited (OR 2.1, 95% CI 1.3–3.5) (Table 1). A total of 64 (45.4%) fetal deaths occurred before admission.

**Table 1:** Characteristics of pregnant cholera patients by pregnancy outcome and risk factors, Haiti, 2011–2014*

Characteristic	No. (%) fetal deaths, n = 141	Unadjusted analysis			Adjusted analysis†	
OR (95% CI)‡	p value§	OR (95% CI)‡	p value§
Age group, y						
<20	30 (21.3)	2.08 (1.30–3.33)	0.01		1.98 (1.21–3.24)	0.02
20–34	96 (68.1)	Referent			Referent	
≥35	15 (10.6)	1.02 (0.57–1.83)			1.00 (0.55–1.84)	
Missing	0					
Time from onset to admission, h						
≤24	59 (41.8)	Referent	0.04		Referent	0.04
>24	82 (58.2)	1.47 (1.02–2.12)			1.49 (1.02–2.17)	
Missing	0					
Trimester of pregnancy						
First	17 (12.1)	Referent	0.02		Referent	0.001
Second	41 (29.1)	1.39 (0.76–2.53)			1.37 (0.74–2.53)	
Third	79 (56.0)	2.08 (1.19–3.64)			2.43 (1.37–4.31)	
Missing	4 (2.8)					
Dehydration level						
None	37 (26.2)	Referent	0.001		Referent	0.02
Moderate	73 (51.8)	1.83 (0.20–2.79)			1.54 (0.99–2.41)	
Severe	31 (22.0)	2.62 (1.55–4.44)			2.17 (1.24–3.81)	
Missing	0					
Vomiting						
Yes	116 (82.3)	2.25 (1.42–3.59)	0.001		2.10 (1.27–3.47)	0.001
No	24 (17.0)	Referent			Referent	
Missing	1 (0.7)					

*Of 900 pregnancies analyzed. OR, odds ratio.  †Adjusted for all list characteristics. ‡From logistic regression model with fetal death as the outcome and list characteristics as exposure. §From Wald tests of parameters of logistic regression model.

Women who experienced preadmission or postadmission fetal death did not differ by age or clinical presentation (Technical Appendix Table 2). However, preadmission fetal death was more likely in women who arrived >24 hours after symptom onset (OR 1.9, 95% CI 0.9–4.1) or were severely dehydrated (OR 2.0, 95% CI 0.7–5.7). In unadjusted analysis, postadmission fetal death was associated with moderate dehydration (OR 2.1, 95% CI 1.2–3.6) and vomiting (OR 2.1, 95% CI 1.2–3.8; Table 2). Of 836 women with a viable fetus at admission, 120 were in TG1, 399 in TG2, and 317 in TG3.

**Table 2:** Characteristics of pregnant cholera patients by pregnancy outcome and treatment group, Haiti, 2011–2014*

Characteristic	No. (%) postadmission fetal deaths, n = 77	Unadjusted analysis		Adjusted analysis†	
OR (95%)‡	p value§	OR (95% CI)†	p value§
Treatment group					
1	12 (15.6)	1.19 (0.58–2.44)	0.85	1.39 (0.66–2.91)	0.68
2	38 (49.4)	1.13 (0.67–1.90)		1.15 (0.67–1.96)	
3	27 (35.1)	Referent		Referent	
Age group, y					
<20	14 (18.2)	1.73 (0.92–3.24)	0.23	1.63 (0.85–3.12)	0.33
20–34	54 (70.1)	Referent		Referent	
≥35	9 (11.7)	1.09 (0.52–2.28)		1.07 (0.50–2.27)	
Missing	0				
Time from onset to admission, h					
≤24	37 (48.1)	Referent	0.57	Referent	0.57
>24	40 (51.9)	1.15 (0.72–1.83)		1.15 (0.71–1.86)	
Missing	0				
Trimester of pregnancy					
First	10 (13.0)	Referent	0.23	Referent	0.06
Second	25 (32.5)	1.44 (0.67–3.08)		1.50 (0.69–3.24)	
Third	41 (53.2)	1.84 (0.90–3.76)		2.28 (1.09–4.76)	
Missing	1 (1.3)				
Dehydration level					
None	20 (26.0)	Referent	0.03	Referent	0.09
Moderate	45 (58.4)	2.08 (1.20–3.61)		1.89 (1.07–3.33)	
Severe	12 (15.6)	1.88 (0.89–3.98)		1.56 (0.71–3.41)	
Missing	0				
Vomiting					
Yes	63 (81.8)	2.10 (1.15–3.82)	0.02	2.05 (1.08–3.90)	0.03
No	14 (18.2)	Referent		Referent	
Missing	0				

*Of 836 women with a viable fetus at admission. OR, odds ratio. †Adjusted for all list characteristics. ‡From logistic regression model with fetal death as the outcome and list characteristics as exposure. §From Wald tests of parameters of logistic regression model.

There was no modification effect of TG on postadmission fetal death. Weak evidence of a difference in effect of severe dehydration on postadmission fetal death between TGs (p = 0.09) (Table 2) was potentially due to a lower rate among severely dehydrated women in TG2 (OR 0.4. 95% CI 0.1–1.7; data not shown). However, there was insufficient power to detect these differences, and the final model did not require adjustment. Although the proportion of postadmission fetal deaths within a TG decreased with each protocol change, the proportion in TG3 (8.5%, 27/317) was not different from TG1 (10.0%, 12/120) or TG2 (9.5%; 38/399).

## Conclusions

Fetal death occurred in 141 (16%) of 900 pregnancies. Risk factors were third trimester, younger maternal age, severe dehydration, and vomiting. Treatment in a specialized CTC and aggressive rehydration did not prevent fetal death though the trend was toward improved outcomes.

Severe dehydration at admission increased risk for fetal death (*2**,**5**−**7*). Fetal death may occur due to fetal hypoxia and acidosis resulting from excessive maternal dehydration (*2**,**4**,**9*). The proportion of fetal deaths we found was higher than that previously recorded in Haiti (8%, 21/263) (*2*) but close to that of the 2006 Senegal cholera outbreak (12%, 6/52) (*3*). Earlier studies in India (*4*), Nigeria (*1**,**7*), and Pakistan (*6*) found higher proportions.

Women <20 years of age were twice as likely as older women to experience fetal death. Although the relationship between fetal death and maternal age during cholera has not been documented, younger age is associated with increased risk for other adverse pregnancy outcomes (*10*). The risk for fetal death was highest in the third trimester, even after controlling for maternal age, dehydration level, and vomiting. The relationship between fetal death and trimester of pregnancy is unclear (*1**,**3**,**6*).

Determination of dehydration status of pregnant women is difficult in later stages of pregnancy (*2**,**11*). Misclassification of dehydration status could affect fetal outcome due to placement of patients under the wrong treatment protocol. In addition, increased placental blood flow with gestational age may increase the effect of dehydration (*12*). Even after we controlled for dehydration level, we determined that fetal death was twice as likely in women experiencing vomiting, potentially due to electrolyte changes in amniotic fluid (*2**,**7**,**13**−**15*).

Lack of effect of a specialized CTC on fetal death could result from a detection bias in that establishment of the specialized CTC led to an increased likelihood of detection of fetal deaths. In addition, 45% of fetal deaths occurred before women sought treatment. Fetal death may occur early in a pregnant woman’s illness with cholera (*6*), and more than half the women sought treatment >24 hours after symptom onset, likely contributing to poor fetal outcomes. Likewise, the effect of the new treatment protocol may have been limited by fetal death occurring before the women sought treatment or by women being assigned the incorrect protocol due to difficulty in determining dehydration status.

Limitations include lack of laboratory-confirmed diagnoses. Data were collected for programmatic rather than research purposes and lack electrolyte levels, amniotic fluid composition, maternal blood group, and fetal cause of death. Some first-trimester pregnancies may have been missed. Pregnancies in women who completed miscarriage at home were not counted, potentially underestimating overall risk for fetal death. Because there was no follow-up of women after discharge, some early-term fetal deaths might have been missed. In addition, the long-term effect of treatment on fetal well-being could not be determined. TG outcomes also may have been affected by differences in factors such as women’s access to health services over time.

Although the implementation of a specialized CTC did not decrease fetal deaths, specialized CTCs play a vital role in preserving patients’ dignity and providing patient-centered care. Determining the mechanism of fetal death in cholera infection would enable development of evidence-based treatment protocols. Because many fetal deaths occurred before women sought treatment, the importance of cholera prevention and the risk for poor fetal outcomes should be emphasized.

Technical Appendix Additional information on treatment protocols and risk factors for pre- and post-admission fetal death among pregnant women with suspected cholera who were admitted to Médecins Sans Frontières’ cholera treatment centers in Port-au-Prince, Haiti, September 1, 2011−December 31, 2014.
