# Supplementary material for: Factors Related to Fetal Death in Pregnant Women with Cholera, Haiti, 2011–2014
Source: Emerg Infect Dis. 2016 Jan;22(1):124–7. doi: 10.3201/eid2201.151078 (PMC4696702; doi:10.3201/eid2201.151078)
Supplement: Technical Appendix — Additional information on treatment protocols and risk factors for pre- and post-admission fetal death among pregnant women with suspected cholera who were admitted to Médecins Sans Frontières’ cholera treatment centers in Port-au-Prince, Haiti, September 1, 2011−December 31, 2014. [file 15-1078-Techapp-s1.pdf]

Article DOI: http://dx.doi.org/10.3201/eid2201.151078

Factors Related to Fetal Death in Pregnant Women with Cholera, Haiti, 2011–2014

Technical Appendix

Table 1. Protocols for treatment of pregnant cholera patients

Variable	Original WHO protocol	New protocol*
Plan A (no dehydration)	ORS: 250ml/stool or as much as the patient can drink	RL: 1000ml over 5 h<br>ORS: 500–750ml per h
Plan B (moderate dehydration)	ORS: 250ml over 15 min	RL: 1000ml over 2.5 h<br>ORS: 750–1000ml per h
Plan C (severe dehydration)	RL: 2000ml over 30 min – repeat if pulse remains weak, otherwise 5000ml over 2.5 h	RL: 1000ml over 15 min – repeat if pulse remains weak, then 1000ml over 45 min, then 1000ml over 2.5 h, then 1000ml over 5 h<br>ORS: 250ml over 15 min
Other treatment	• Erythromycin – 500mg 4x/day for 3 d<br>• D/W 50% 20ml in 1000ml RL over 2 h for hypokalemia<br>• APE: 20–60mg furosemide depending on edema, repeat after 6hrs if required then reduce to 20mg each morning until edema is stabilized.<br>• If vomiting is incoercible insert a nasogastric tube	• Azithromycin −1g single dose<br>• D/W 50% 20ml in 1000ml RL over 2 h for hypokalemia<br>• APE: 20–60mg furosemide depending on edema, repeat after 6h if required then reduce to 20mg each morning until edema is stabilized.<br>• If vomiting is incoercible insert a nasogastric tube
Corticotherapy	• For all women 24–36 weeks: 6mg dexamethasone every 12h, 4 injections	• For all women 24–36 weeks: 6mg dexamethasone every 12h, 4 injections
Fetal heart rate	• Checked each hour (or each 15 min during active phase of labor, and each 30 min latent phase of labor).	• Checked each hour (or each 15 min during active phase of labor, and each 30 min latent phase of labor).
Birth	• Vulva wash with 0.05% chlorine solution, rinse with water<br>• Keep amniotic sac intact as long as possible<br>• Avoid the presence of any stool in the environment at time of birth.	• Vulva wash with 0.05% chlorine solution, rinse with water<br>• Wash newborn with 0.05% chlorine solution<br>• Keep amniotic sac intact as long as possible<br>• Avoid the presence of any stool in the environment at time of birth.

ORS = oral rehydration salts. RL = lactated Ringer solution. D/W = dextrose in water. APE = acute pulmonary edema.
*New treatment protocol implemented June 1, 2013. There were three treatment groups: TG1 = general cholera treatment center (CTC) + original treatment protocol (admitted September 1, 2011 to March 31, 2012); TG2 = specialized CTC + original treatment protocol (April 1, 2012 to May 31, 2013); TG3 = specialized CTC + new treatment protocol (June 1, 2013 to December 31, 2014).

Table 2. Number (%) of fetal deaths by risk factors (n = 141)

Characteristic	Pre-admission fetal death, n = 64	Post-admission fetal death, n = 77	Unadjusted analysis OR (95%CI)†	Unadjusted analysis p‡	Fetal death: pre- vs post-admission* OR (95%CI)†	Fetal death: pre- vs post-admission* p‡
Age group, y						
<20	16 (25.0)	14 (18.2)	1.47 (0.65–3.35)	0.60	1.93 (0.78–4.75)	0.30
20–34	42 (65.6)	54 (70.1)	Referent		Referent	
≥35	6 (9.4)	9 (11.7)	0.86 (0.28–2.60)		0.79 (0.25–2.54)	
Missing	0 (0.0)	0 (0.0)				
Time from onset to admission						
≤24 h	22 (34.4)	37 (48.1)	Referent	0.10	Referent	0.08
>24 h	42 (65.6)	40 (51.9)	1.77 (0.89–3.49)		1.94 (0.93–4.05)	
Missing	0 (0.0)	0 (0.0)				
Trimester of pregnancy						
First	7 (10.9)	10 (13.0)	Referent	0.61	Referent	0.56
Second	16 (25.0)	25 (32.5)	0.91 (0.29–2.89)		0.69 (0.20–2.43)	
Third	38 (59.4)	41 (53.2)	1.32 (0.46–3.83)		1.08 (0.35–3.35)	
Missing	3 (4.7)	1 (1.3)				
Dehydration level						
None	17 (26.6)	20 (26.0)	Referent	0.11	Referent	0.06

Characteristic	Pre-admission fetal death, n = 64	Post-admission fetal death, n = 77	Unadjusted analysis OR (95%CI)†	Unadjusted analysis p‡	Fetal death: pre- vs post-admission* OR (95%CI)†	Fetal death: pre- vs post-admission* p‡
Medium	28 (43.8)	45 (58.4)	0.73 (0.33–1.63)		0.64 (0.27–1.54)	
Severe	19 (29.7)	12 (15.6)	1.86 (0.71–4.91)		1.98 (0.68–5.71)	
Missing	0 (0.0)	0 (0.0)				
Vomiting						
Yes	53 (82.8)	63 (81.8)	1.18 (0.48–2.87)	0.72	1.32 (0.49–3.51)	0.58
No	10 (15.6)	14 (18.2)	Referent		Referent	
Missing	1 (1.6)	0 (0.0)				

Data are presented as no. (%) unless otherwise specified
OR = odds ratio. CI = confidence interval.
*Adjusted for all list characteristics
†From logistic regression model with fetal death as the outcome and list characteristics as exposure
‡From Wald tests of parameters of logistic regression model
